# Green Tea Extract Treatment Alleviates Ocular Inflammation in a Rat Model of Endotoxin-Induced Uveitis

**DOI:** 10.1371/journal.pone.0103995

**Published:** 2014-08-05

**Authors:** Yong Jie Qin, Kai On Chu, Yolanda Wong Ying Yip, Wai Ying Li, Ya Ping Yang, Kwok Ping Chan, Jia Lin Ren, Sun On Chan, Chi Pui Pang

**Affiliations:** 1 Department of Ophthalmology and Visual Sciences, The Chinese University of Hong Kong, Hong Kong; 2 School of Biomedical Sciences, The Chinese University of Hong Kong, Hong Kong, China; National Eye Institute, United States of America

## Abstract

Green tea extract (GTE) ingested by rats exerted anti-oxidative activities in various ocular tissues as shown in our previous studies. The present work investigated anti-inflammatory effects of GTE on endotoxin-induced uveitis (EIU). EIU was generated in adult rats by a footpad injection of 1 mg/kg lipopolysaccharide (LPS). Oral administration of GTE (550 mg/kg) was given one, two or four times after LPS injection. Twenty-four hours later, LPS produced severe hyperemia and edema in the iris. Immunocytochemical examinations showed an accumulation of infiltrating cells in the aqueous humor that were immunopositive for cluster of differentiation 43 (CD43) and CD68, markers for leucocytes and macrophages, respectively. Analyses of the aqueous humor showed an increase in pro-inflammatory mediators including tumor necrosis factor-alpha (TNF-α), interleukin-6 (IL-6) and monocyte chemoattractant protein-1 (MCP-1). GTE treatments improved the clinical manifestations and reduced infiltrating cells and protein exudation in the aqueous humor, which were not observed under half dose of GTE (275 mg/kg). The number of CD68 positive macrophages residing in the iris and ciliary was also reduced. GTE suppressed production of TNF-α, IL-6 and MCP-1 in the aqueous humor, which was associated with a down-regulation of LPS receptor complex subunits, Toll-like receptor 4 (TLR-4) and CD14, and suppression of nuclear factor-kappa Bp65 (NF-κBp65) in the iris and ciliary body. Our findings show that GTE is a potent anti-inflammatory agent against the inflammation of EIU, and suggest a potential use in treatment of acute uveitis.

## Introduction

Uveitis, an ocular inflammatory condition, accounts for approximately 10–15% cases of total blindness and up to 20% of legal blindness in developed world [1], [2]. The goal of uveitis treatment is to suppress inflammation and achieve regression when it occurs [3]. However inflammation can recur with various complications, such as cataract and permanent cumulative damages [4], [5]. Administrations of corticosteroid are standard therapeutic strategy, but they have many potential side effects such as intraocular pressure increase, cataract formation and increase in infection susceptibility [6]. Therefore, alternative treatments which are safer and more long lasting are needed.

The rat model of endotoxin-induced uveitis (EIU) has been widely used for evaluating potential ocular anti-inflammatory compounds since it was reported in 1980 [7]–[12]. EIU can be induced by systemic injection of lipopolysaccharide (LPS), which generates inflammatory responses largely in the anterior uvea and mild responses in the posterior segments of the eye, mimicking the pathological conditions in human acute uveitis [12]–[14]. It has been reported that LPS was recognized by membrane-bound cluster of differentiation 14 (mCD14) and Toll-like receptors (principally TLR-4) on the surface of macrophages. Receptors activation in these immune surveillance cells resulted in phosphorylation of nuclear factor-kappa B (NF-κB) and caused release of pro-inflammatory factors, such as tumor necrosis factor-α (TNF-α), interlukin-6 (IL-6) and monocyte chemoattractant protein-1 (MCP-1) [15]–[21]. As a crucial proximal mediator, TNF-α stimulates acute phase reaction of inflammation by influencing leukocyte activation and infiltration, and inducing production of other mediators such as IL-6, a major cytokine regulator of acute phase response [22], [23]. MCP-1, a powerful chemotactic and activating factor [24], stimulates the activation of mitogen-activated protein kinases (MAPKs) to promote monocytes migration [25]. Another study shows that the blood-humor barrier is broken down two hours after LPS injection [26], which results in migration of polymorphonuclear and mononuclear cells into the aqueous humor. The inflammatory process reaches peak level at 18–24 hours after LPS injection [27].

Catechins, the major component in green tea extract (GTE), have been shown to exert anti-oxidative, anti-inflammatory, anti-angiogenic and anti-carcinogenic effects [28], [29]. In a pharmacokinetic study, we have shown that catechins reach peak level in the ocular tissues of normal rats at 1 to 2 hours after ingestion, and produce significant reduction in oxidative stress within these tissues [30]. We have addressed in this study the hypothesis that orally administered GTE could serve as effective anti-inflammatory agents to alleviate inflammatory responses in anterior segments of the eye triggered by a systemic injection of LPS.

## Materials and Methods

### Endotoxin-induced uveitis and GTE treatment

All experiments were conducted according to the Association for Research in Vision and Ophthalmology (ARVO) statement on the use of animals. Ethics approval for this study was obtained from the Animal Ethics Committee of the Chinese University of Hong Kong. Sprague-Dawley rats (about 250 g, 6–8 weeks old) were obtained from the Laboratory Animal Service Center of the Chinese University of Hong Kong. Ethics approval for this study was obtained from the Animal Ethics Committee of the University. All animals were housed at 25°C with 12/12 hour light-dark cycles, and were allowed to access freely to food and water. Before the experiment, animals were fasted overnight and body weight was recorded.

EIU was induced by injection of 0.1 mL of pyrogen-free saline dissolved LPS (from Salmonella typhimurium; Sigma Chemical, St. Louis, MO, USA) at the dose of 1 mg/kg into one footpad. The dosage was selected according to results of a preliminary study, which showed that LPS at 1 mg/kg was the optimal dose in inducing moderate inflammation in both eyes without causing obvious lesion in the liver and kidney. The GTE Theaphenon E was kindly provided by Dr. Y. Hara, which contains EGCG (epigallocatechin gallate, >65%), EGC (epigallate catechins, <10%), EC (epicatechin, <10%) and ECG (epicatechin gallate, <10%) and other trace catechin derivatives. It was prepared as a 550 mg/kg GTE suspension in 0.5 mL distilled water and was fed intragastrically into the rat.

The rats were randomly divided into three treatment groups: i) GTE1, fed with GTE two hours after LPS injection (LPS+GTE1, n = 6); ii) GTE2, fed with GTE twice at two and eight hours after LPS injection (LPS+GTE2, n = 6); iii) GTE4, fed with GTE four times at two, five, eight and eleven hours after LPS injection (LPS+GTE4, n = 6). Control groups consisted of: i) normal control, footpad injected with saline and fed with water two hours after injection (Saline+water, n = 3); (ii) LPS controls, footpad injected with LPS and fed with water (LPS+water, n = 6); (iii) Dxm controls, footpad injected with LPS and fed with Dexamethasone (Dxm) (1 mg/kg, distilled water suspension; Sigma Chemical, USA) two hours after LPS injection (LPS+Dxm, n = 6); and (iv) GTE controls, footpad injected with saline and fed with GTE four times as in GTE4 group (Saline+GTE4, n = 3). Another eighteen rats were used for histological studies.

In another experiment, the dosage effect of GTE was tested in 23 rats: i) normal control (n = 5), footpad injected with saline followed by oral administration of water at the 2^nd^ and 11^th^ hours after footpad injection; ii) LPS group (n = 6), footpad injection of LPS followed by feeding of water at the 2^nd^ and 11^th^ hour after footpad injection; iii) 550 mg/kg GTE (n = 6), oral administration of the dose at the 2^nd^ and 11^th^ hour after footpad injection of LPS; iv) half dose GTE (n = 6), oral administration of 275 mg/kg GTE at the 2^nd^ and 11^th^ hour after the LPS injection.

Twenty four hours after LPS injection, the rats were anesthetized with intraperitoneal injection of 4.0 mL of ketamine-xylazine mixture (1.5∶1, Alfasan International B.V., Holland) for collections of ocular tissues. They were terminated immediately by drawing the whole blood through heart puncture.

### Clinical manifestations scoring

Clinical features of ocular inflammation in both eyes were evaluated using a slit lamp and graded from score 0 to score 4 by a masked observer 24 hours after LPS injection as described previously [31]. The grading is assigned as: 0 = no obvious inflammatory response; 1 = discrete dilation of iris and conjunctival vessels; 2 = moderate dilation and iris and conjunctival vessels with moderate flare in the anterior chamber; 3 = intense iridal hyperemia with intense flare in the anterior chamber; 4 = same clinical signs as 3 with presence of fibrinoid exudation and miosis.

### Histological examination of infiltrating cells

Under deep anesthesia, the rats were perfused intracardially with 0.01 M sterile phosphate buffer saline (PBS) followed by 4% paraformaldehyde. Both eyes were removed and immersed in 10% formalin for 24 hours at room temperature. Some connective tissues were maintained on each eye to facilitate orientation. The eyes were embedded in paraffin and sectioned in 5 µm thickness along the vertical meridian and the optic nerve head. After deparaffinization and rehydration, the sections were stained with Hematoxylin and Eosin (H&E). The anterior and posterior segments of the eyeball were examined under a light microscope (DMRB, Leica Microsystems, Wetzlar, Germany). Total number of infiltrating cells located in anterior segments (anterior/posterior chamber) and posterior segments (vitreous body around optic nerve head) were counted in a masked fashion by an ocular pathologist as described previously [32].

Some sections were selected for immunostaining in order to determine the identity of infiltrating cells. The slides were heat to induce epitope retrieval using a pressure cooker (Biocare Medical, Walnut Creek, CA). After blocking with 0.1% bovine serum at room temperature, mouse anti-rat monoclonal antibody CD43 (1∶80 dilution, AbD Serotec, Kidlington, UK) or CD68 (1∶100 dilution, AbD Serotec, Kidlington, UK) was applied separately to the sections and incubated at 4°C overnight, which binds preferentially to leucocytes and macrophages, respectively. The sections were then washed and incubated in secondary antibody (1∶1000 dilution; Alexa Fluor 488 for CD43, Alexa Fluor 594 for CD68; Invitrogen, Carlsbad, CA, USA) at room temperature for 1 hour. DAPI (4′,6-diamidino-2-phenylindole, 1∶2000 dilution) was used for counter-stain of nuclei. The sections were mounted using aqueous mounting medium (GBI Labs, Manchester, UK) and examined under fluorescence microscope (Diagnostic Instruments, Sterling Heights, Michigan). Control sections were processed as above without primary antibody.

In another experiment, sections of the eye were processed for immunostaining of CD68 in order to determine whether GTE treatment reduced LPS-induced accumulation of macrophages in the stroma of iris and ciliary body. Four non-consecutive sections were collected from the eye of normal controls, and animals treated with LPS alone, LPS plus GTE or dexamethasone. The number of CD68 positive cells inside stroma of iris and ciliary body was counted in a masked fashion.

### Cell count and protein assay in aqueous humor

Aqueous humor was collected by piercing the anterior chamber with a 30 gauge needle. One microliter aqueous humor was diluted with 9 µl 0.01 M PBS and suspended in an equal volume of Trypan-blue solution. The number of cells was counted by using a hemacytometer under a light microscope. Another portion of the aqueous humor was centrifuged at 2500 rpm for 15 minutes at 4°C. Cells-free supernatant was used for total protein assay in duplicate (Bio-Rad, Hercules, CA, USA).

### Determination of pro-inflammatory factors in the serum and aqueous humor

Blood samples were collected by heart puncture and clotted at room temperature for 2 hours in serum vial. They were centrifuged at 2500 rpm for 15 minutes at 4°C. The serum and cells-free aqueous humor were taken for determination of TNF-α and IL-6 (rat-ELISA kit, R&D Systems, Minneapolis, USA) and MCP-1 (rat-ELISA kit, Invitrogen, Camarillo, CA, USA), each in duplicate.

### Quantification of CD14 and TLR-4 mRNA expression

Iris, ciliary body/process and retina were collected. After washing with 0.01 M cold sterile PBS, tissues were immersed in 300–500 µl Trizol reagent (Invitrogen, USA) and stored at −80°C until use. Total RNA were isolated and treated with RNase-free DNaseI according to the manufacturer’s protocol (Qiagen, Hilden, Germany). RNA (0.5–1 µg) was reversely transcribed into cDNA using SuperScript III reverse transcriptase (Invitrogen, USA). PCR was performed using iCycler PCR instrument (Bio-Rad) and lightCycle 480 II real-time PCR system (Roche Applied Science, Penzberg, Germany). Sequences of the gene-specific primers were designed using the online Primer 3 Input Program (version 0.4.0) (Table 1).

**Table 1:** Primer sequences used for quantitative real-time PCR detection of rat-CD14, TLR-4 and GAPDH.

Gene	Identification	Forward, 5′-3′	Reverse, 5′-3′	Product size
*CD14*	NM_021744.1	TCACAATTCACTGCGGGATA	CGATGTCCTAGGAGCAAAGC	330 bp
*TLR-4*	NM_019178.1	TGATGCCTCTCTTGCATCTG	TCCAGCCACTGAAGTTGTGA	247 bp
*GAPDH*	NM_017008.4	GTGCCAGCCTCGTCTCATA	GTTGAACTTGCCGTGGGTAG	190 bp

CD14: cluster of differentiation 14; TLR-4: toll-like receptor 4; GAPDH: glyceraldehyde-3-phosphate dehydrogenase.

Quantitative real-time PCR (qPCR) was performed with SYRB green PCR mixture containing 10 µl of 2×480 SYRB green I Master (Roche, USA), 0.55 µl of 10 nM primers (Invitrogen), 1 µl cDNA and 8 µl double distilled water. The parameters were as follows: pre-incubation 95°C for 10 minutes, followed by 40 amplification cycles each with denaturation at 95°C for 15 seconds, annealing/extension at 60°C for 1 minute. The melt-curve analysis was performed using default settings of the instrument. Final qPCR products were electrophoresed in 2.0% agarose gel. Relative CD14 and TLR-4 mRNA expression of each sample was calculated as described [33]. In brief, after the threshold cycle value of the target gene (C_T_ *target*) was emendated with the value of internal control gene GAPDH (C_T *GAPDH*_), the normalized expression value (2^−(CT *target*−CT *GAPDH*)^) was calculated, then the expression fold change due to treatment were obtained according to the normalized expression value.

### Western blot analysis

Nuclear proteins were isolated from iris and ciliary body by using ReadyPrepTM Protein Extraction Kit (Cytoplasmic/Nuclear, Bio-Rad, Hercules, CA, USA). Protein concentration was adjusted equally with protein assay kit (Bio-RAD, Hercules, CA, USA), then re-suspended in 5x sample loading buffer, heated for 5 min at 95°C and separated on 12.5% SDS-polyacrylamide gel electrophoresis (SDS-PAGE). The proteins were transferred onto a nitrocellulose membrane (AmershamTM HybondTM-ECL, GE Healthcare, UK), blocked with 5% Bovine albumin BSA (A9418, Sigma-Aldrich, USA), and incubated with NF-κBp65 antibody (1∶300, sc-372, Santa Cruz Biotechnology, INC.) and Lamin B antibody (1∶500, sc-6216, Santa Cruz Biotechnology, INC.) at 4°C overnight. After washing with TBS-0.05% tween-20 (TBST), HRP-coupled secondary antibodies (1∶1000, Santa Cruz Biotechnology, INC.) were applied to the membrane for 1 hour at room temperature, followed by three washes with TBST. The immunoreactive bands were visualized with enhanced chemiluminescence reagents (GE Healthcare, UK) and images were captured by the Universal Hood II image system (Bio-Rad Laboratories, Segrate, Italy). Band intensities of NF-κBp65 were normalized with those of internal control (Lamin B) using NIH Image J software (version 1.47).

### Statistical analysis

All data were analyzed by nonparametric Kruskal-Wallis Tests. Two group comparisons were done by Mann-Whitney Tests. The *p*-value less than 0.05 was considered statistically significant. All statistical analyses were performed using SPSS statistical software package (version 20.0).

## Results

### Clinical manifestations of inflammation in the eye

**Figure 1:**
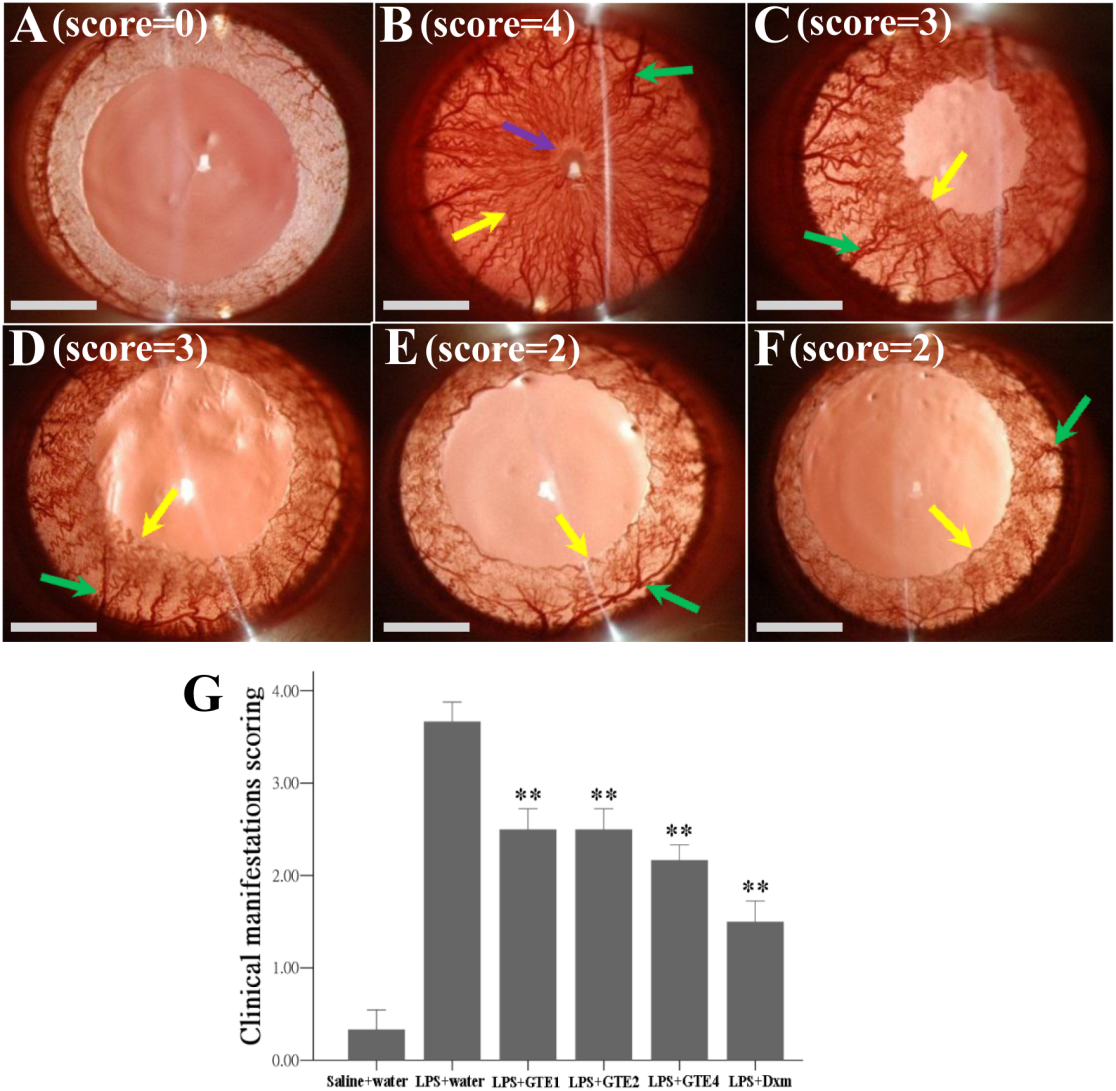
Clinical manifestations of ocular inflammation in rat eyes. Ocular inflammation was evaluated by slit lamp examination 24 hours after LPS injection. (A): No inflammatory feature was observed in normal control rats (saline+water). (B) and (C): Hyperemia (green arrow), edema (yellow arrow) and synachesia (purple arrow) occurred in the iris of LPS treated rats. (D) and (E): Inflammatory responses were subsided in rats treated with GTE1 (D) and GTE4 (E). (F): Inflammatory responses were also suppressed in rats treated with Dexamethasone (Dxm). (G): The scores of clinical features were reduced significantly after GTE and Dxm treatments (**p<0.05, when compared with LPS+water). Saline+water, n = 3; LPS+water, n = 6; LPS+GTE1, n = 6; LPS+GTE2, n = 6; LPS+GTE4, n = 6; LPS+Dxm, n = 6. Data were shown as mean ± *SE*. Scar bar = 2 mm.

Slit lamp examination showed development of hyperemia and edema associated with miosis and fibril formation in the iris 24 hours after LPS injection (Fig. 1B–C). These clinical features were not observed in the normal controls (Fig. 1A), and appeared less severe in the GTE treated rats (Fig. 1D–E) and rats treated with dexamethasone (Fig. 1F). Quantitative evaluation of these clinical scores showed a significant reduction in animals treated with 1 to 4 times oral administration of GTE (*p*<0.05) when compared with animals fed with water after LPS insult (Fig. 1G).

### Infiltrating cells in anterior segments of the eye

**Figure 2:**
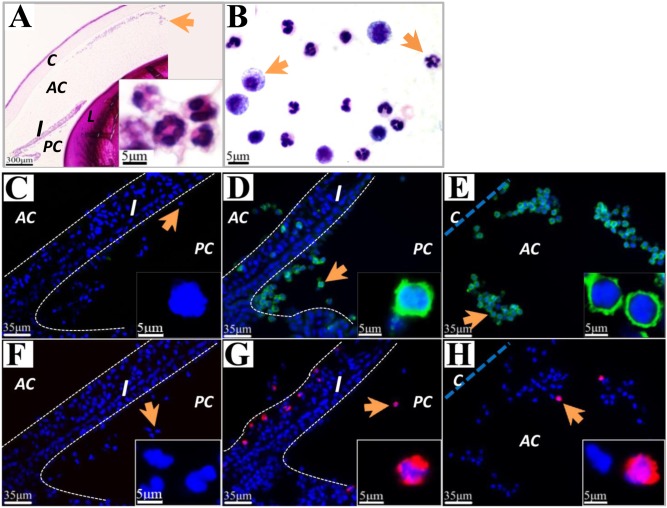
Histological features of infiltrating cells in ocular tissues. Paraffin sections showed infiltrating cells in the anterior segments of the eye after LPS insult. (A): H&E section showed clusters of infiltrating cells (arrow, magnified in the insert) with polymorphic nuclei in anterior (AC) and posterior chamber (PC). (B): Giemsa staining of infiltrating cells (arrows) in aqueous humor. (D) and (E): Fluorescent micrographs showing localization of CD43 on leukocytes (arrow, magnified in the insert) in iris (I), PC and AC. (G) and (H): CD68 was localized on macrophage/monocytes (arrows, magnified in the insert) in iris, PC and AC. No staining was observed in control sections treated without primary antibody against CD43 (C) or CD68 (F). C: cornea.

Injection of LPS induced substantial accumulation of infiltrating cells in the anterior chamber and posterior chamber of the eye (Fig. 2A). Giemsa staining showed that these infiltrating cells in aqueous humor were variable in size and polymorphic in nuclear shape (Fig. 2B). Immunohistochemical studies on paraffin sections of LPS treated eye showed that most infiltrating cells in anterior and posterior chamber were CD43 positive leucocytes (Fig. 2D–E), whereas a few were CD68 positive macrophages (Fig. 2G–H). A few CD43 and CD68 positive cells were found in the stroma of iris and ciliary body (Fig. 2D and G). No staining was detected in sections processed with the absence of primary antibody (Fig. 2C and F).

**Figure 3:**
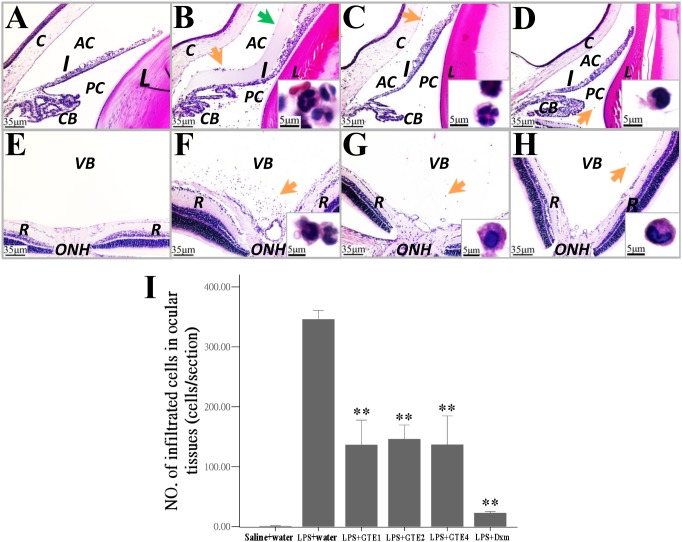
The number of infiltrating cells in ocular tissues after GTE treatments. (A), (B), (C) and (D): H&E sections showing accumulation of infiltrating cells (orange arrows, magnified in inserts) and protein exudation (green arrow) in anterior segments of the eye. (E), (F), (G) and (H): H&E sections showing infiltrating cells (arrows) in vitreous body (VB) around the optic nerve head (ONH). (A, E): Saline+water; (B, F): LPS+water; (C, G): LPS+GTE4; (D, H): LPS+Dxm. (I): The number of infiltrating cells in the anterior and posterior segments of the eye was reduced in rats treated with GTE (**p<0.05) when compared with that with LPS and vehicle treatment. n = 3 in each group. Data were shown as mean ± *SE*. L: lens; CB: ciliary body; R: retina.

The effects of GTE treatment on ocular inflammation were investigated in paraffin sections of the eye. LPS induced an accumulation of infiltrating cells and proteineous substances in the anterior and posterior chamber (Fig. 3B), which was not observed in normal control animals (Fig. 3A). Treatment of GTE reduced substantially the infiltration of cells and accumulation of protein in the anterior and posterior chamber (Fig. 3C), which was further reduced in animals treated with Dxm (Fig. 3D). Similar observations were presented in their corresponding vitreous body around the optic nerve head (Fig. 3E–H), where the accumulation of infiltrating cells was highly reduced in both GTE (Fig. 3G) and Dxm (Fig. 3H) treated animals. Counting of infiltrating cells on these sections (in anterior and posterior chamber, and in vitreous space) showed a significant reduction in GTE treated animals (*p*<0.05) when compared with the vehicle treated LPS group (LPS+water) (Fig. 3I).

### Cell infiltration and protein exudation in aqueous humor

**Figure 4:**
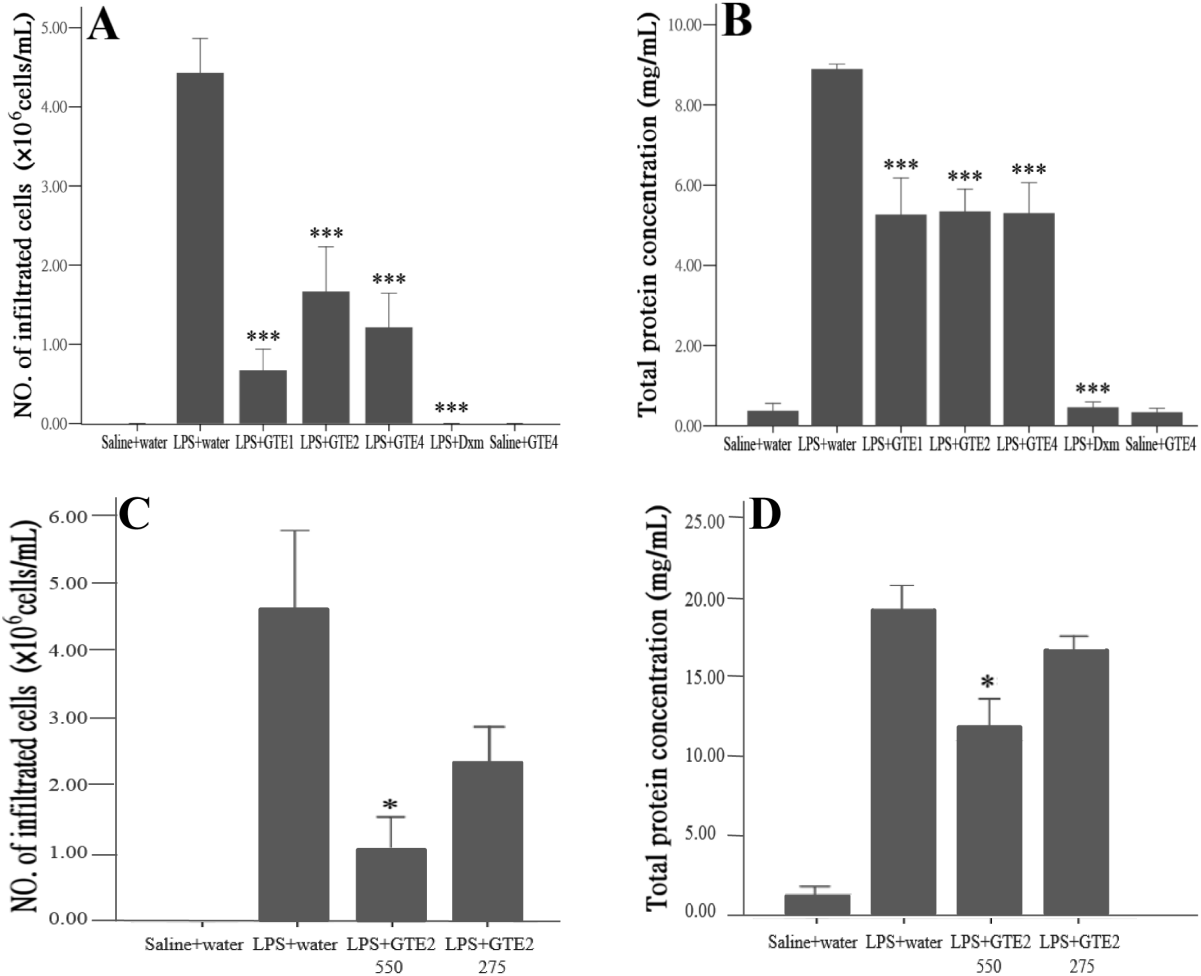
GTE treatment on cell infiltration and protein exudation in aqueous humor. (A) and (B): GTE caused a significant reduction in accumulation of cells (A) and protein exudation (B) in aqueous humor (***p<0.01) when compared with LPS+water. The differences among the GTE treatment groups were, however, not significant (*p*>0.05). Saline+water, n = 3; LPS+water, n = 6; LPS+GTE1, n = 6; LPS+GTE2, n = 6; LPS+GTE4, n = 6; LPS+Dxm, n = 6; Saline+water, n = 3. (C) and (D): In another experiment, significant reduction in infiltrating cells (C) and protein content (D) in aqueous humor was observed only in rats treated with 550 mg/kg GTE (*p<0.05), but not in those received half dose (275 mg/kg) of GTE. Saline+water, n = 5; n = 6 in LPS+water, LPS+GTE2 550 and LPS+GTE2 275. Data were shown as mean ± *SE*.

The changes in infiltrating cells and protein content in aqueous humor were also analyzed. Treatments of GTE produced significant decrease in infiltration cells (*p*<0.05) and accumulation of protein (*p*<0.05) when compared with vehicle treated LPS group (Fig. 4A–B), agreeing with the results in histological analyses, though these effects were not as potent as Dxm (*p*<0.01). No differences were observed among animals treated with one, two or four times of oral administrations of GTE, agreeing with the findings in the histological study. The reason of lack of effect for multiple dosage was unclear, but may be caused by the rapid decline of anti-oxidative property of GTE in the aqueous humor [30]. Treatment with GTE alone without injection of LPS produced neither an accumulation of cells nor an increase in protein exudation in the aqueous humor.

The dosage effect was tested in another experiment, in which the rats were fed with either 550 or 275 mg/kg GTE, 2 and 11 hours after LPS injection. The results showed that both infiltrating cell number and protein level in aqueous humor were significantly reduced at the dose of 550 mg/kg (*p*<0.05), but the effect became insignificant when the dose was reduced to half (Fig. 4C–D), indicating that 550 mg/kg is an effective dose in suppressing these inflammatory responses.

### TNF-α, IL-6, and MCP-1 in aqueous humor

**Figure 5:**
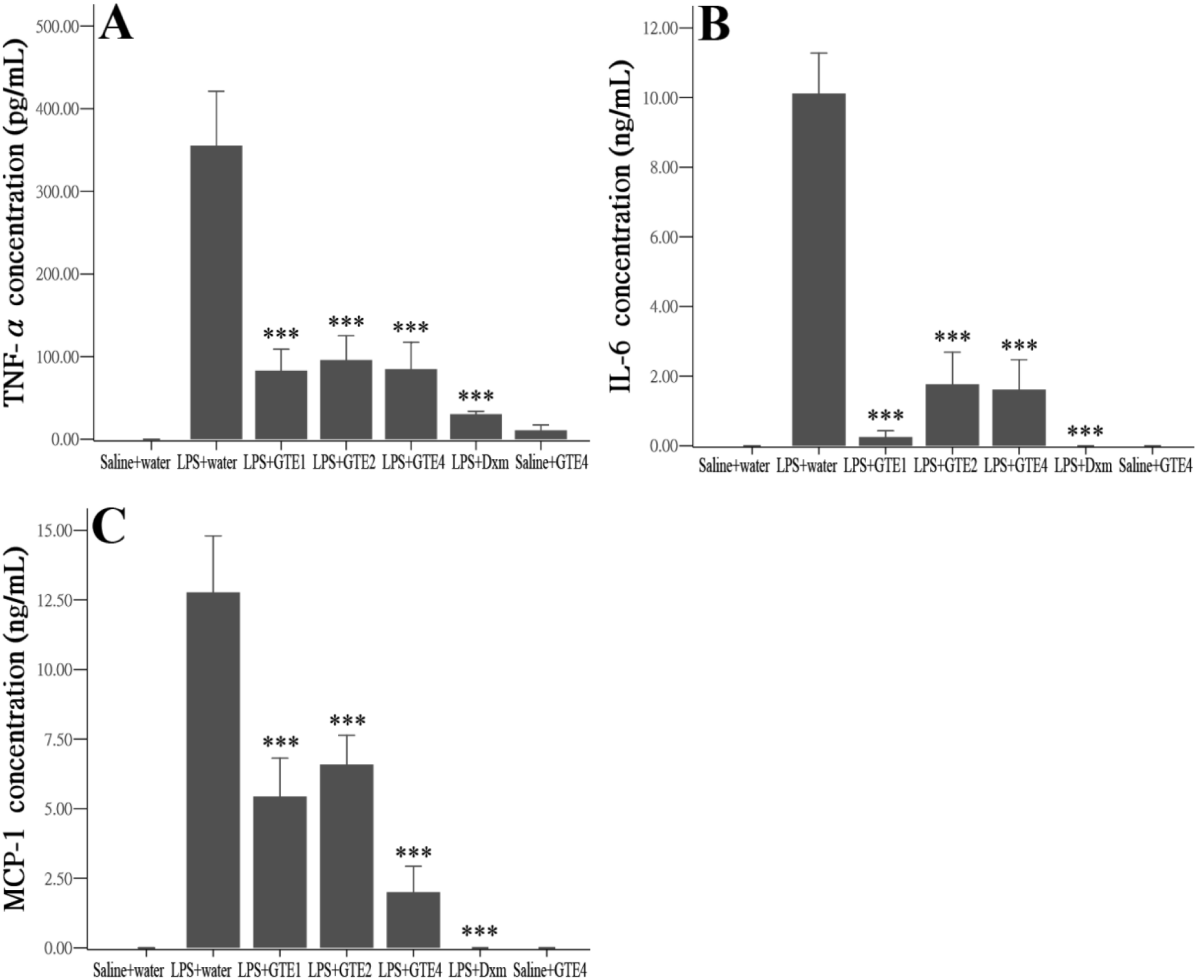
Effects of GTE on cytokine and chemokine production in aqueous humor. The pro-inflammatory factors TNF-α (A), IL-6 (B) and MCP-1 (C) were all reduced significantly after GTE treatments when compared with LPS+water (***p<0.01). Within group comparisons, however, did not show obvious differences among GTE treated groups (*p*>0.05). Sensitivity of the assays: 5 pg/mL for TNF-α, 21 pg/mL for IL-6, less than 8.0 pg/mL for MCP-1. Saline+water, n = 3; LPS+water, n = 6; LPS+GTE1, n = 6; LPS+GTE2, n = 6; LPS+GTE4, n = 6; LPS+Dxm, n = 6; Saline+water, n = 3. Data were shown as mean ± *SE*.

The pro-inflammatory factors TNF-α, IL-6 and MCP-1 were determined in the aqueous humor using ELISA. LPS caused a surge in TNF-α, IL-6 and MCP-1, which were barely detected in normal controls (Fig. 5A–C). All GTE treatments caused a significant reduction of these factors when compared with the LPS group (LPS+water) (*p*<0.05). However, no significant difference was detected within the GTE groups (*p*>0.05), suggesting that multiple treatments did not produce additional effect in reducing these pro-inflammatory factors. The effect of Dxm was more potent than both GTE1 and GTE2 only for MCP-1 (GTE1 vs Dxm, *p* = 0.007; GTE2 vs Dxm, *p* = 0.002) but not for TNF-α and IL-6. GTE alone did not produce any detectable change in these factors. Analyses of the serum showed no significant reduction of pro-inflammatory factors in animals treated with either GTE when compared with those treated with LPS and vehicle (Table 2). Dexamethasone treatment caused a significant reduction in MCP-1 (*p*<0.01) but not in IL-6. While TNF-α was beyond the detectable level in the serum, both GTE and Dxm treatments could not reduce the level of IL-6 and MCP-1 to that of normal control.

**Table 2:** TNF-α, IL-6 and MCP-1 concentration in the serum after different treatments.

	Serum, mean ± *SD*				
Groups	TNF-α (pg/mL)	IL-6 (ng/mL)	*p* value	MCP-1 (ng/mL)	*p* value
Saline+water (n = 3)	-	-	-	-	-
LPS+water (n = 6)	-	0.16±0.15	-	6.19±1.19	-
LPS+GTE1 (n = 6)	-	0.12±0.10	0.749	4.82±0.67	0.078
LPS+GTE2 (n = 6)	-	0.08±0.06	0.378	5.20±1.40	0.337
LPS+GTE4 (n = 6)	-	0.13±0.10	0.936	4.59±1.15	0.055
LPS+Dxm (n = 6)	-	0.05±0.08	0.121	1.74±2.07**	0.004
Saline+GTE4 (n = 3)	-	-	-	-	-

-: undetectable. Sensitivity of these assays is 5 pg/mL for TNF-α, 21 pg/mL for IL-6, less than 8.0 pg/mL for MCP-1.

Saline+water: saline injection and fed with water; LPS+water: LPS injection and fed with water; LPS+GTE1: GTE was fed once after LPS injection; LPS+GTE2: GTE was fed twice after LPS injection; LPS+GTE4: GTE was fed four times after LPS injection; LPS+Dxm: Dxm was fed once after LPS injection; Saline+GTE4: saline injection and fed as GTE4; **p<0.05, compared with LPS+water.

### Expression of CD14 and TLR-4 mRNA, and NF-κBp65 activity in ocular tissues

**Figure 6:**
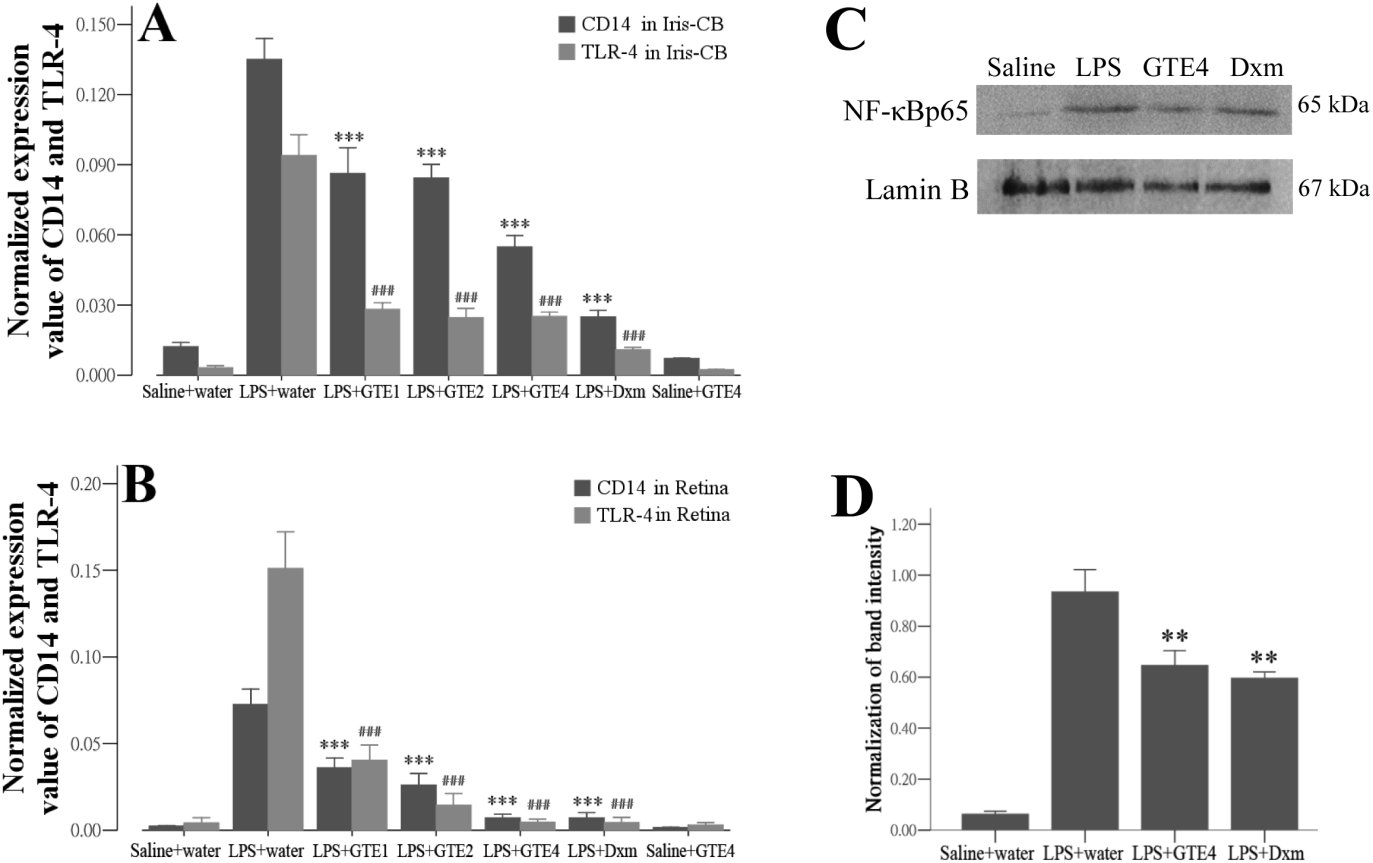
Effect of GTE on CD14 and TLR-4 mRNA expression and nuclear NF-κBp65 protein expression in ocular tissues. (A) and (B): Quantitative PCR analyses showing expression of CD14 and TLR-4 transcripts in the iris and ciliary body (A), and the retina (B). Both genes were reduced significantly after GTE or Dxm treatment, when compared with the values in LPS+water group. GTE treatments alone (saline+GTE4) did not generate obvious changes to these expression genes. Saline+water, n = 3; LPS+water, n = 6; LPS+GTE1, n = 6; LPS+GTE2, n = 6; LPS+GTE4, n = 6; LPS+Dxm, n = 6; Saline+GTE4, n = 3. (C): Nuclear proteins isolated from iris and ciliary body were analyzed using Western blot to detect the level of nuclear NF-κBp65. Lamin B was used as internal control. (D): Measurements of band intensity of NF-κBp65/Lamin B showed a significant reduction of nuclear NF-κBp65 after treatment with GTE or dexamethasone (Dxm). n = 3 in each plot. Data were shown as mean ± *SE*. **p<0.05, *** and ###: *p<*0.01, compared with LPS+water.

Quantitative real-time PCR was used to determine the expression of TLR-4 and CD14, the respective receptor and co-receptor for LPS, after LPS injection and GTE treatment (Table 3). Expression of both TLR-4 and CD14 were up-regulated after LPS insult in the iris and ciliary body (Fig. 6A) and in the retina (Fig. 6B). These elevated expressions were suppressed significantly by GTE or Dxm treatment (*p*<0.01, Fig. 6A–B). GTE treatment alone without LPS injection did not caused obvious changes in TLR-4 and CD14 expression when compared with normal control.

**Table 3:** CD14 and TLR-4 mRNA expression in the iris and ciliary body and the retina by quantitative real-time PCR analyses.

	Iris-ciliary body					Retina				
	C_T_ values, mean ± *SD*	Fold change in expressiondue to treatment				C_T_ values, mean ± *SD*			Fold change in expressiondue to treatment	
Groups	*CD14*	*TLR-4*	*GAPDH*	*CD14*	*TLR-4*	*CD14*	*TLR-4*	*GAPDH*	*CD14*	*TLR-4*
Saline+water (n = 3)	24.93±0.36	26.42±0.69	18.28±0.32	−11	−31	27.18±0.18	27.12±1.15	18.50±0.60	−37	−38
LPS+water (n = 6)	21.38±0.96	21.54±0.36	18.28±0.70	1	1	22.37±.74	20.84±0.29	18.51±0.42	1	1
LPS+GTE1 (n = 6)	22.65±0.51	24.32±0.44	19.09±0.30	−2	−3	22.25±1.12	23.10±0.90	17.84±1.12	−2	−4
LPS+GTE2 (n = 6)	22.23±1.31	23.57±0.51	18.39±0.97	−2	−4	25.00±1.44	25.81±0.76	18.97±0.81	−2	−11
LPS+GTE4 (n = 6)	22.92±0.83	24.05±0.29	18.71±0.48	−2	−4	25.49±1.90	27.06±1.43	17.80±0.42	−10	−30
LPS+Dxm (n = 6)	23.99±0.52	25.18±0.50	18.63±0.32	−5	−9	29.04±2.37	25.50±2.37	19.60±1.69	−10	−38
Saline+GTE4 (n = 3)	26.21±0.40	27.26±0.38	18.82±0.48	−19	−47	27.45±0.71	27.43±2.27	18.15±0.56	−37	−50

C_T_: threshold cycle; Saline+water: saline injection and fed with water; LPS+water: LPS injection and fed with water; LPS+GTE1: GTE was fed once after LPS injection; LPS+GTE2: GTE was fed twice after LPS injection; LPS+GTE4: GTE was fed four times after LPS injection; LPS+Dxm: Dxm was fed once after LPS injection; Saline+GTE4: saline injection and fed as GTE4.

The level of nuclear NF-κBp65, a downstream molecule of TLR-4 signaling, was examined in the iris and ciliary body after LPS and GTE treatments. Western blot showed that this protein was upregulated in the tissues after LPS injection, and its expression was decreased substantially after treatment of GTE4 and Dxm (Fig. 6C). Measurements of band intensity with reference to that of Lamin B confirmed a significant reduction of nuclear NF-κBp65 after GTE4 or Dxm treatment (*p*<0.05) (Fig. 6D).

### Effect of GTE on macrophages within iris and ciliary body

**Figure 7:**
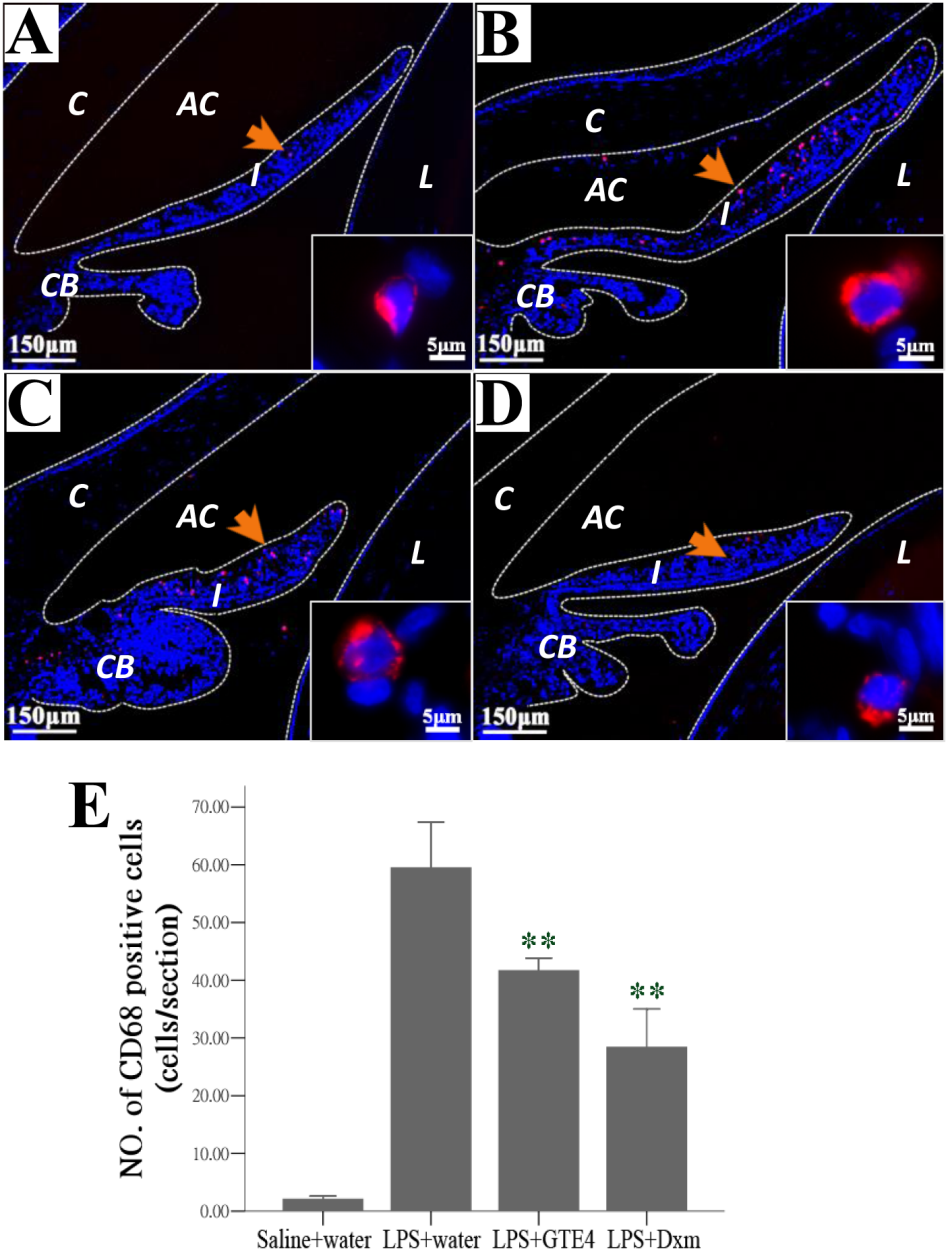
Effect of GTE on macrophages within iris and ciliary body. (A)–(D): Fluorescent micrographs showing some cells were immunopositive to CD68, a marker for macrophages (arrows, magnified in inserts) 24 hours after treatment with (A): saline+water, (B): LPS+water, (C): LPS+GTE4, (D): LPS+Dxm. (E): A significant reduction in number of macrophages presented in the stroma of iris and ciliary body was observed after treatment with GTE and dexamethasone (Dxm). n = 3 in each group. Data were shown as mean ± *SE*. **p<0.05 compared with LPS+water. C: cornea; AC: anterior chamber; I: iris; PC: posterior chamber; L: lens; CB: ciliary body.

To investigate the effect of GTE treatment on the macrophages accumulating in the iris and ciliary body, sections of the eye were immunostained with antibody against CD68. In normal control only a few CD68 positive macrophages were observed in the stroma of iris and ciliary body (Fig. 7A). The number of macrophages was increased substantially after LPS treatment (Fig. 7B and E), and was reduced significantly after treatment of either GTE4 or Dxm (*p*<0.05) (Fig. 7C–E).

## Discussion

Results of this study show that oral administration of GTE, at the dose of 550 mg/kg, produces anti-inflammatory effects against LPS-induced ocular inflammation. The major findings include: i) GTE alleviates clinical manifestations of ocular inflammation; ii) it reduces infiltration of leukocytes and macrophages, and leakage of protein into aqueous humor and vitreous body; iii) it also suppresses production of pro-inflammatory biomarkers TNF-α, IL-6 and MCP-1 in aqueous humor; iv) these anti-inflammatory effects are associated with down-regulation of LPS receptors, TLR-4 and CD14, and reduction of nuclear NF-κBp65, v) GTE also reduces accumulation of macrophages in the stroma of iris and ciliary body. These results provide evidence for the first time that GTE is a potent anti-inflammatory agent for acute ocular inflammation.

We have shown in this study that the levels of pro-inflammatory mediators, TNF-α, IL-6 and MCP-1, were elevated significantly in the aqueous humor twenty-four hours after LPS injection. TNF-α has been shown in rats to be the major player in LPS-induced leukocyte adhesion, vascular leakage and cell death in the rats’ eyes [34]. In mice deficient of MCP-1, inflammatory responses are reduced in a model of EIU [32]. The surge of infiltrating leukocytes and macrophages in the aqueous humor is thus likely caused by an increased production of pro-inflammatory factors as a response to the LPS insult. LPS also induces an accumulation of macrophages in the stroma of iris and ciliary body, either by recruitment of circulating macrophages or activation of resident cells in these tissues. GTE exerts its anti-inflammatory actions through a suppression of production of these pro-inflammatory factors, and thereby reducing the infiltration of leucocytes and macrophages and exudation of protein into the aqueous humor, and recruitment or activation of macrophages residing in the iris and ciliary body. These anti-inflammatory effects seem to be specific to ocular tissues as shown by the results that significant reduction of IL-6 and MCP-1 after GTE treatment is observed only in the aqueous humor but not in the serum. The specific changes agree with previous findings that TNF-α, IL-6 and MCP-1 are elevated in aqueous humor of patients with infectious or noninfectious uveitis but not in the serum [35], [36]. Reduction of TNF-α was detected only in the aqueous humor but not in the serum, possibly because its properties of brief production [37] and high hepatic clearance [38] in the circulatory system.

Juxtaposition of CD14 and TLR-4 in the anterior uvea contributes to the sensitivity of iris and ciliary body to LPS [39]. CD14 does not have a trans-membrane segment and so needs TLR-4, a trans-membrane protein, to transduce the LPS stimulation. LPS binds primarily to resident macrophages or epithelial cells lining the iris and ciliary processes [39], [40]. When LPS-CD14-TLR4 cluster activation is inhibited by lipid raft-disrupting drug or lipid A mimetic antagonists, LPS-induced cellular cascading reaction is interrupted [41], [42]. We have shown in this study that GTE suppresses LPS induced elevation of CD14 and TLR-4 mRNA in the anterior uvea and the retina, suggesting that GTE treatment may alleviate ocular inflammation by suppressing formation of the LPS receptor complex. These findings are consistent with previous reports showing that EGCG is able to block the interaction between LPS and TLR-4 and downregulate TLR-4 mRNA expression *in* *vitro* [43], [44]. However, other receptor components, such as HSP 70/90 (heat-shock proteins 70 and 90), CXCR4 (chemokine receptor 4), and DAF (decay-accelerating factor) also participate in the LPS-CD14-TLR4 cluster activation, probably acting as additional LPS-transfer molecules [45]. Moreover, EGCG has also been shown to suppress inflammatory responses by binding to the surface molecule 67-kDa laminin receptor (76LR), which results in reduction of expression of pro-inflammatory mediators, such as TNF-α, IL-6 and cyclooxygenase-2 [43]. Whether GTE may modulate function of these molecules remains to be investigated.

Anti-inflammatory properties of GTE have been reported in other experimental disease models. Epigallocatechin-3-gallate (EGCG), the major constituent of GTE, has shown to reduce expression of inflammatory biomarkers in human cell lines such as chondrocytes and corneal epithelium, and experimental models of dry eye [46]–[48]. These effects are related to a suppression of IκB kinase-β (IKKβ) and TANK-binding kinase-1 (TBK1) [49], or inhibition of the proteasome-mediated degradation pathway that induces accumulation of NF-κB inhibitors, p27^Kip1^ and IκB-α [50]. We have shown in the current study that LPS induced nuclear NF-κBp65 is significantly reduced after GTE treatment, probably caused by a reduced level of TLR-4-CD14 receptor complex, and leads eventually to a reduced expression of genes coding for cytokines and chemokines.

To our knowledge, this is the first report to demonstrate anti-inflammatory effects of GTE on acute ocular inflammation. The findings in this study support strongly that GTE is a potent therapeutic agent for treatment of acute anterior uveitis.
